# The role of the farnesoid X receptor in kidney health and disease: a potential therapeutic target in kidney diseases

**DOI:** 10.1038/s12276-023-00932-2

**Published:** 2023-02-03

**Authors:** Dong-Hyun Kim, Jung Sun Park, Hoon-In Choi, Chang Seong Kim, Eun Hui Bae, Seong Kwon Ma, Soo Wan Kim

**Affiliations:** Department of Internal Medicine, Chonnam National University Medical School, Gwangju, 61469 Korea

**Keywords:** End-stage renal disease, Acute kidney injury

## Abstract

The prevalence of kidney diseases has been increasing worldwide due to the aging population and has results in an increased socioeconomic burden as well as increased morbidity and mortality. A deep understanding of the mechanisms underlying the physiological regulation of the kidney and the pathogenesis of related diseases can help identify potential therapeutic targets. The farnesoid X receptor (FXR, NR1H4) is a primary nuclear bile acid receptor that transcriptionally regulates bile acid homeostasis as well as glucose and lipid metabolism in multiple tissues. The roles of FXR in tissues other than hepatic and intestinal tissues are poorly understood. In studies over the past decade, FXR has been demonstrated to have a protective effect against kidney diseases through its anti-inflammatory and antifibrotic effects; it also plays roles in glucose and lipid metabolism in the kidney. In this review, we discuss the physiological role of FXR in the kidney and its pathophysiological roles in various kidney diseases, including acute kidney injury and chronic kidney diseases, diabetic nephropathy, and kidney fibrosis. Therefore, the regulatory mechanisms involving nuclear receptors, such as FXR, in the physiology and pathophysiology of the kidney and the development of agonists and antagonists for modulating FXR expression and activation should be elucidated to identify therapeutic targets for the treatment of kidney diseases.

## Kidney disease: role of key bile acid receptor

Targeting the expression and activation of a critical bile acid receptor could be a novel therapeutic approach for kidney diseases. The prevalence of all types of kidney disease is rising due to ageing populations, but there are few therapeutic options available. In a review of recent research, Dong-Hyun Kim, Soo Wan Kim and co-workers at Chonnam National University in Gwangju, South Korea, examined the functional role of a key bile acid receptor protein called farnesoid X (FXR) in health and disease. FXR is widely expressed in the liver, intestines and kidneys, where it maintains bile acid and cholesterol levels, and exerts anti-inflammatory and anti-fibrotic effects. However, existing studies of its role are largely limited to the liver and intestines. The team call for further research into its function in different cell types in the kidney.

## Introduction

The kidney is one of the important organs that maintains body homeostasis by excreting waste, regulating acid–base and electrolyte balance, and modulating the pressure, composition, and volume of blood. Additionally, the kidneys have an endocrine function; they produce and activate several hormones important for calcium and phosphorus metabolism.

Acute kidney injury (AKI), characterized by a rapid decline in kidney function, results in significant morbidity, mortality, and high medical costs; it can progress to chronic kidney disease (CKD) or end-stage kidney disease (ESKD)^1^. Despite the increasing incidence of kidney disease and medical costs worldwide, AKI and CKD have no specific treatment, and dialysis and kidney transplantation are the only treatments currently available for ESKD. Therefore, the basic physiological and pathophysiological mechanisms in the kidney should be elucidated for the development of new and effective therapeutic strategies, and nuclear receptors should be explored.

This review focuses on the functional role of the farnesoid X receptor (FXR, NR1H4) in kidney homeostasis and kidney diseases. FXR belongs to the nuclear receptor family, which has 48 members in humans and is activated by bile acids. This receptor plays a key role in maintaining bile acid and cholesterol levels and is highly expressed in the liver, intestine, and kidneys^2,3^. This review summarizes the recent studies on the roles of kidney-specific FXR in health and diseases and discusses the development of potential therapeutics.

### Overview of FXR

FXR was initially described as a farnesol-activated receptor that interacts with the retinoid X receptor (RXR) and was later found to be activated by bile acids^4,5^. Humans and mice have four FXR isoforms (FXRα1, FXRα2, FXRα3, and FXRα4), resulting from the use of different promoters and alternative splicing between exons 5 and 6^6,7^. However, the cellular and physiological functions of these FXR isoforms are unclear. RNA sequencing of microdissected rat kidneys and immunofluorescence staining of kidneys from wild-type (WT) and FXR knockout (KO) mice have shown that FXR is mainly expressed in the proximal tubules, specifically in the initial segment of the proximal tubule (PTS1) and the proximal straight tubules in cortical medullary rays (PTS2). FXR is also expressed in other kidney regions, including the distal tubules, thin descending limbs, and collecting ducts^8–10^.

The endogenous ligands of FXR are conjugated and unconjugated bile acids; the ligand pocket is dependent on the bile acid type with the following ranking of potency: chenodeoxycholic acid (CDCA) > deoxycholic acid (DCA) > lithocholic acid (LCA) >> cholic acid (CA)^4,5,11^. Synthetic FXR agonists, including steroidal and nonsteroidal ligands, for the treatment of liver diseases, such as nonalcoholic steatohepatitis (NASH) and primary biliary cholangitis (PBC), are currently under development. Ligand-activated FXR binds to FXR response elements, such as inverted repeats (IR0 and 1), directed repeats (DR1, 2, 4, and 5), and everted repeats (ER2 and 8), as a monomer or heterodimer with RXR in the promoter or introns of target genes^12–15^. Although deep sequencing after chromatin immunoprecipitation (ChIP-seq) is a powerful technique to identify the genome-wide binding sites of transcription factors in the liver and intestine, this technique has not yet been performed to study FXR in the kidney. Furthermore, FXR has been largely studied in the liver and intestines, and its roles in kidney physiology and diseases have been recently pursued.

### Role of FXR in kidney physiology and pathophysiology

In this review, we focus on the roles of FXR in kidney physiology and pathophysiology, including its roles in regulating urine concentration, water volume, and lipid metabolism; we also describe its involvement in vascular diseases, AKI, CKD, and diabetic nephropathy (DN). This review aims to summarize the functional roles of kidney-specific FXR and the protective effects of FXR agonists in various kidney diseases (Table 1).

**Table 1:** Summary of the role of FXR in the physiology and pathophysiology of the kidneys.

Target disease	Treatment	Experimental model	Description and function	Refs.
Urine volume and osmolality	CDCA	- C57BL/6	AQP2, urine osmolality ↑, Urine Vol. ↓	^8^
	CDCA GW4064	- C57BL/6 - mIMCD3	TonEBP, cell viability ↑	^19^
	CDCA GW4064	- CD-FXR KO - MCDs	CRYZ ↑, cell death ↓, BCL2 ↑	^20^
Lipid metabolism	GW4064 CA	- C57BL/6 J - HFD - C57BLKS/J-*db/db* - Mouse mesangial cells	Fatty acid synthesis, fibrosis, glomerulosclerosis ↓, inflammation, oxidative stress ↓	^32^
	OCA (INT-747)	- DBA/2 J - WD	Fatty acid synthesis, fibrosis ↓ Inflammation, oxidative stress ↓ Triglyceride accumulation ↓	^33^
Vascular disease and CKD	OCA (INT-747) INT-767	- ApoE KO-CKD(5/6 nephrectomy) - CVCs	Calcification, MSX2, osterix ↓, p-JNK ↑	^37^
	OCA (INT-747) PX20606 DCA	- LDLR KO-CKD (5/6 nephrectomy) - DBA/2J-CKD (5/6 nephrectomy) - FXR KO; LDLR KO - CKD (5/6 nephrectomy)	Serum DCA level, ATF4, ER stress ↓ CKD-dependent-medial calcification & atherosclerotic calcification ↓	^39^
	OCA (INT-747)	- Rat - CKD (0.75% Adenine) - HASMCs	Vascular calcification ↓, miR-135a-5p ↑, TGFBR1/TAK1 signaling ↓	^40^
AKI	OCA (INT-747)	- C57BL/6J - I/R injury - PTCs	ROS & ER stress, inflammation & apoptosis ↓, GSH & NRF2 ↑	^43^
	ABA (alisol B 23-acetate)	- C57BL/6 & FXR KO - I/R injury - HK-2, mTECs, mIMCD3	Inflammation, apoptosis, ROS ↓	^44^
	OCA (INT-747)	- ICR mice-LPS	NF-κB activity ↓, glutathione depletion & lipid peroxidation↓, NADPH oxidation genes ↑	^45^
	GW4064 OCA (INT-747) WAY-362450	- C57BL/6 & FXR KO - I/R injury - HK-2 cells	Autophagy & apoptosis related genes ↓, ROS ↓, progression of AKI to CKD ↓	^50^
	OCA (INT-747)	- C57BL/6 - cisplatin - HK-2 cells	Inflammation, apoptosis, & fibrosis ↓, kidney injury ↓	^52^
	GW4064	- C57BL/6, FXR KO, FXR ^flox/flox^, PT-FXR KO, OT-FXR KO - cisplatin - PTECs	Fatty acid oxidation & PPARγ signaling related genes ↑, lipid accumulation ↓	^10^
	GW4064	- C57BL/6 & FXR KO -cisplatin - HK-2 cells	Ferroptosis, lipid peroxidation, iron level ↓, GSH/GSSG ↑, GPX4 & AIFM2 (FSP1) ↑	^55^
	Dioscin	- Rat - Doxorubicin - NRK-52E cells	Oxidative stress & inflammation ↓, NF-κB activity & HMGB ↓	^56^
CKD	GW4064 CDCA	- C57BL/6J - UUO - HK-2 & HKC cells	Fibrosis ↓, SMAD3 expression ↓	^60^
	EDP-305	- C57BL/6 - UUO - HK-2 cells	Interstitial fibrosis ↓, p-YAP & YAP nuclear localization ↓	^63^
	GW4064 WAY-362450	- C57BL/6J & FXR KO -UUO - HK-2 & NRK49F cells	Fibrosis, inflammation ↓, p-Src kinase ↓, p-YAP & YAP nuclear localization ↓	^62^
Diabetic nephropathy (DN)	GW4064	- HMCs - high glucose	TGFβ/SMAD signaling, inflammatory response, visfatin ↓	^71^
	OCA (INT-747)	- C57BL/6 & FXR KO - STZ & WD - DBA/2J - STZ & WD	Proteinuria, glomerulosclerosis, tubulointerstitial fibrosis, oxidative stress, SREBPs ↓	^72^
	OCA (INT-747) INT-767 INT-777	- DBA/2J - STZ & WD - C57BLKS/J-*db/db* - C57BL/6J - HFD	Lipogenesis, mitochondria biogenesis, inflammation, oxidative stress, fibrosis ↓	^73^
	TUDCA	- C57BLKS/J-*db/db* - eNOS KO - STZ - Podocytes & HKC-8 cells	SOCS3, DDAH1 ↑, glomerular and tubular injury ↓	^76^
	GW4064	- C57BL/6J - tacrolimus (FK506) - HK-2 cells	Gluconeogenesis ↓, glucose uptake ↑	^80^

*CDCA* chenodeoxycholic acid, *mIMCD3* inner medullary collecting duct cell line, *MCDs* medullary collecting duct cells, *CD-FXR KO* collecting duct-specific FXR knockout, *HFD* high-fat diet, *WD* Western-style diet, *OCA* obeticholic acid (INT-747; 6-ECDCA: 6α-ethyl-chenodeoxycholic acid), *CVCs* bovine calcifying vascular cells, *DCA* deoxycholic acid; *LDLR KO* low-density lipoprotein receptor knockout mice, *FXR KO* farnesoid X receptor knockout mice, *VSMCs* human vascular smooth muscle cells, *HASMCs* human aortic smooth muscle cells, *I/R injury* ischemia/reperfusion injury; *PTCs* primary cultured proximal tubular cells, *mTECs* mouse proximal tubular epithelial cells, *LPS* lipopolysaccharide; *PTECs* proximal tubular epithelial cells, *UUO* unilateral ureteral obstruction, *HMCs* human mesangial cells, *STZ* streptozotocin, *HKC-8* human proximal tubular epithelial cells.

#### FXR function in urine concentration and water regulation

One of the important functions of the kidneys is the modulation of urine volume and osmolality. The urine volume of FXR-deficient mice is higher and their osmolality is lower than those of WT mice. Conversely, FXR activation by endogenous (CDCA and CA) or synthetic (GW4064) agonist treatment increases the expression of aquaporin 2 (AQP2) in mouse kidneys and cultured primary inner medullary collecting duct (IMCD) cells; it also decreases urine volume while increasing urine osmolality^8^. AQP2 is an aquaporin channel protein expressed in the principal cells of the kidney collecting ducts. It controls urine concentration, and its expression is tightly regulated by its transcriptional activator arginine vasopressin (AVP) and antidiuretic hormone. However, in studies on Takeda G protein-coupled receptor 5 (TGR5)/FXR signaling, the activation of FXR alone by CDCA or obeticholic acid (OCA, INT-747, or 6α-ethyl-chenodeoxycholic acid) does not upregulate AQP2 in a mouse model of lithium-induced nephrogenic diabetes insipidus; conversely, the activation of TGR5 by INT-777 (TGR5 agonist) or INT-767 (FXR and TGR5 dual agonist) increases AQP2 expression through the TGR5-activated cAMP-protein kinase A (PKA) signaling pathway^16^. TGR5 is a member of the G protein-coupled receptor family for bile acids and is involved in bile acid homeostasis, glucose and lipid metabolism, and inflammatory responses^17^. In addition to AVP, hypertonicity in the kidney medulla is critical in controlling urine concentration. The medullary collecting duct cells (MCDs) of FXR-deficient mice undergo massive apoptosis under hypertonic stress, and treatment with FXR agonists (CDCA and GW4064) or FXR overexpression in the mouse inner medullary collecting duct cell line (mIMCD3) increases the expression and activity of nuclear factor of activated T-cell 5 (TonEBP, also called NFAT5), which is a critical transcription factor for the osmotic activation of kidney medullary cells under hyperosmotic stress^18,19^. Crystallin zeta (CRYZ), which participates in the stabilization of Na^+^/K^+^/2Cl^-^ cotransporter mRNA in the kidney, is a direct target gene of FXR. The expression of *CRYZ* is regulated by FXR activation in MCDs, and *CRYZ* overexpression attenuates hypertonicity-induced cell death by increasing B-cell lymphoma 2 (BCL2) expression^20^. These data demonstrate the critical role of FXR in the regulation of urine volume and osmolality by modulating the expression of proteins in the MCDs of the kidneys.

Another important function of the kidneys is the regulation of fluid volume and blood pressure homeostasis by sodium reabsorption. The epithelial sodium channel (ENaC) regulates sodium transport and sodium balance in the collecting duct and functions as a major component that regulates blood pressure homeostasis^21^. Although studies have not yet explored the direct regulation of ENaC by FXR, studies have explored the activation and inhibition of human and murine ENaC by bile acids^22,23^. In addition, nitric oxide (NO) plays an important role in blood pressure control, and impaired NO bioactivity is a crucial factor in hypertension^24^. Bile acids or bile acid receptor agonists attenuate hypertension by improving vascular reactivity through the regulation of NO bioactivity. In vascular endothelial cells, treatment with CDCA and GW4064 upregulates the mRNA and protein expression levels of endothelial nitric oxide synthase (eNOS) and increases NO production^25^. In spontaneously hypertensive rats, CDCA treatment attenuates the increased blood pressure and stimulates eNOS compared with vehicle treatment^26^. In a recent study, C57BL/6 mice with hypertension induced by 20% fructose in drinking water with 4% sodium chloride (HFS) in the diet for 8 weeks had increased blood pressure; they also had decreased renal NO levels and FXR expression. Intrarenal FXR overexpression in HFS-fed mice and FXR agonist treatments in mIMCD-K2 cells attenuate hypertension and promote NO production by regulating the expression of dynamin 3 (DNM3), which is a binding protein with neuronal NOS in the renal medulla^27^. Overall, studies have been conducted on sodium modulation by bile acids and blood pressure regulation by FXR agonists; however, further studies are needed to evaluate the mechanisms and therapeutic potential of FXR and bile acid receptors.

#### FXR function in lipid metabolism

FXR is known as a key regulator of cholesterol and lipid homeostasis, and several studies have reported the correlation between kidney dysfunction and kidney lipid accumulation in various disease models, including metabolic disease (obesity, metabolic syndrome, and diabetes mellitus), AKI, and CKD^28^.

In diabetes and diet-induced obesity mouse models, triglyceride and cholesterol levels are increased compared with the levels in healthy controls. These increased kidney triglyceride levels are associated with an increase in the levels of sterol regulatory element-binding protein 1c (SREBP-1c) and carbohydrate response element-binding protein (chREBP), two transcription factors that increase fatty acid synthesis^29^. SREBP-1c is a critical factor that increases fatty acid synthesis and lipid accumulation in the kidneys^30,31^. In these two models, treatment with FXR agonists shows a kidney-protective effect by downregulating the genes related to fatty acid synthesis, including SREBP-1c, stearoyl-CoA desaturase-1 (SCD-1), and acetyl-CoA carboxylase (ACC), and by upregulating the genes related to fatty-acid oxidation and lipid catabolism, including peroxisome proliferator-activated receptor α (PPARα), carnitine palmitoyl transferase 1a (CPT1a), uncoupling protein-2 (UCP-2), peroxisome proliferator-activated receptor-γ coactivator-1α (PGC-1α), and lipoprotein lipase (LPL)^32,33^.

#### FXR function in vascular diseases

When apolipoprotein E (ApoE) KO mice and low-density lipoprotein receptor (LDLR) KO mice are treated with an FXR agonist (OCA) or FXR/TGR5 dual agonist (INT-767), atherosclerosis, aortic plaque formation, and the levels of circulating cytokines, including IL-1b, IL-6, IL-8, and IL-12, decrease via inactivation of NF-κB^34,35^. To investigate the roles of inactivated FXR and TGR5 in vivo, Miyazaki-Anzai et al. generated LDLR KO mice lacking FXR and TGR5 expression^36^. FXR and TGR5 deficiency in LDLR KO mice completely blocks the antiatherogenic and anti-inflammatory effects of INT-767 and causes severe atherosclerosis and aortic inflammation^36^. Thus, FXR has been studied as a therapeutic target to reduce vascular inflammation and atherosclerotic plaque stability, and vascular disease is one of the complications of kidney diseases.

In studies exploring the correlation between CKD and vascular disease, FXR agonist (OCA) treatment of ApoE KO mice with CKD (5/6 nephrectomized) and bovine calcifying vascular cells (CVCs) prevented CKD-induced calcification by inhibiting the expression of osteogenic transcription factors (msh homeobox 2; MSX2 and osterix) through the activation of c-Jun N-terminal kinase (JNK)^37^. In addition, severe vascular calcification in patients with stage 3-4 CKD is associated with high serum bile acid levels in these patients, particularly their free DCA levels; FXR/TGR5 dual agonist (INT-767) treatment in mice reduces the levels of CA and CA-derived bile acids, such as DCA^35,38^.

In CKD, endoplasmic reticulum (ER) stress-mediated activating transcription factor-4 (ATF4), a major factor that induces vascular calcification, is expressed and activated by DCA. The FXR-deficient CKD mouse model has significantly higher ER stress because of the higher DCA level and severe vascular calcification than the WT levels. Activation of FXR by agonists (OCA and PX20606) inhibits the transcription of enzyme-encoding genes such as CYP8B1 and CYP7A1 involved in CA-DCA synthesis. Consequently, serum DCA levels, ER stress, and ATF4 activity decrease, thereby reducing vascular calcification^39^. In CKD rat models and human aortic smooth muscle cells (HASMCs) cultured with osteogenic medium, FXR activation increases the expression of miR-135a-5p, which inhibits the transforming growth factor-β (TGFβ) receptor 1 (TGFBR1)/TGF-β-activated kinase 1 (TAK1) pathway and attenuates vascular calcification^40^.

#### Role of FXR in acute kidney injury

AKI, also known as acute renal failure, is a sudden decline in kidney function that occurs within a few days. It is associated with CKD because it can cause irreversible nephron loss and shorten the kidney lifespan^41^. Ischemic/reperfusion (I/R) injury and pathological pathways, such as the release of reactive oxygen species (ROS), cytokines, and inflammatory mediators and the activation of neutrophils, are involved in the pathogenesis of AKI^42^.

The FXR agonist OCA has been shown to protect the kidneys from I/R injury by alleviating the associated oxidative stress, including the restoration of the decrease in intracellular glutathione (GSH) levels and the increase in ROS levels, through upregulation of nuclear factor erythroid-2-related factor 2 (NRF2)-mediated antioxidant genes^43^. In addition, the novel natural FXR agonist alisol B 23-acetate (ABA), extracted from Alismatis Rhizoma, attenuates increased kidney inflammation, apoptosis, and ROS formation in an I/R-induced AKI mouse model^44^.

Lipopolysaccharide (LPS) is involved in the pathogenesis of sepsis-induced AKI and has been shown to cause an inflammatory response and upregulate ROS. In mice, the FXR agonist OCA has a protective effect against LPS-induced AKI by blocking the nuclear translocation of nuclear factor kappa B (NF-κB, p65, and p50 subunits), restoring glutathione depletion and increasing NADPH oxidase^45^. The exact mechanism in the kidney is not known, but in a study on posttranslational modifications (PTMs) of FXR within the liver, FXR affected NF-κB activity. Under physiological conditions, agonist-activated FXR is modified by SUMO2 at K277, and SUMO2-FXR is a selective transrepressor of inflammatory genes by increasing its interaction with NF-κB^46^.

Autophagy is a cellular homeostatic process through which damaged organelles and dysfunctional components are removed and cellular proteins are recycled through the lysosomal degradation pathway. It is associated with several diseases and occurs according to the response to internal and external cellular stresses. In the AKI model, the role of autophagy has been studied in the inflammation, apoptosis, and mitochondrial damage that occurs during AKI, kidney interstitial fibrosis and progressive CKD^47^.

Feeding and fasting regulate the transcription of hepatic autophagy by FXR-cAMP-response element binding protein (CREB) and FXR-PPARα^48,49^. In the kidneys, autophagy genes can be regulated by not only FXR agonists (GW4064 and OCA) but also feeding and fasting in vivo. In addition, the increased autophagy- and apoptosis-related genes and ROS levels in the I/R injury mouse model and hypoxia-induced HK-2 cells were decreased by FXR activation. Autophagy should be tightly controlled to protect against the progression of AKI to CKD. The levels of ROS and autophagy-related proteins were restored to the control levels in WT mice 7 and 28 days after I/R, respectively, unlike in the FXR-deficient mouse model. Thus, FXR plays an important role in the regulation of kidney autophagy during the progression of AKI to CKD^50^.

Cisplatin is the most widely used therapeutic agent for the treatment of solid-organ cancer; however, it causes nephrotoxicity as a side effect. Although the mechanism of cisplatin-induced AKI is unclear, it may be related to changes in cellular signaling, the cell cycle, and associated cell death pathologies^51^.

FXR agonist (OCA) treatment suppresses the increases in proinflammatory signaling (TNF-α and IL-1β) and the levels of cell-surface adhesion molecules (MCP-1 and ICAM-1) in the cisplatin-induced AKI mouse model by activating the transcription of a small heterodimer partner (SHP; NR0B2), which is known as an FXR target gene. In addition, OCA attenuates cisplatin-induced apoptosis and tubular atrophy by inhibiting the MAPK pathway^52^. In a comparative study on cisplatin-induced AKI in WT and FXR KO mice, the levels of serum creatinine, blood urea nitrogen, and kidney tubular injury in the FXR KO-cisplatin group were significantly higher than those in the WT-cisplatin group; furthermore, FXR deficiency aggravated cisplatin-induced AKI, possibly by inhibiting autophagy and promoting apoptosis^53^.

By crossing Kap-Cre (Cre recombinase under the control of the kidney androgen-regulated protein promoter) mice with FXR^flox/flox^ mice, Xu et al. generated FXR^flox/flox^ Kap-Cre mice, whose FXR is specifically knocked out in kidney proximal tubules (PTs)^10^. These animals revealed the importance of FXR localization during cisplatin-induced AKI. PT cells are highly energized primarily through fatty acid oxidation (FAO). RNA sequencing of primary mouse proximal tubular epithelial cells (PTECs) overexpressing FXR has revealed that the expression of the genes associated with FAO and peroxisome proliferator-activated receptor-γ (PPARγ) signaling is upregulated. The activation and overexpression of FXR specifically in PTs reduce lipid accumulation during cisplatin-induced AKI by improving FAO by activating the transcription of *PPARγ* and carnitine palmitoyltransferase (*CPT1*), a rate-limiting enzyme in FAO^10^.

Cisplatin may increase lipid accumulation, oxidative stress, lipid peroxidation, and free fatty acid levels, which are associated with ferroptosis. Ferroptosis is a type of nonapoptotic cell death program mediated by iron-dependent lipid peroxidation^54^. Glutathione peroxidase 4 (GPX4, the central regulator of ferroptosis) and FXR were decreased in a cisplatin-induced AKI mouse model compared with the levels in healthy controls. Additionally, FXR KO mice show increased malondialdehyde (MDA, a marker of lipid peroxidation) and iron levels and a decreased GSH/GSH disulfide (GSH/GSSG) ratio compared with those of WT mice. Notably, FXR agonist treatment in cisplatin-induced AKI mice alleviated kidney injury and the ferroptotic response, whereas FXR deficiency exacerbated these pathological features of AKI. RNA sequencing and ChIP assays have revealed that FXR regulates the transcription of ferroptosis-associated genes, including apoptosis-inducing factor mitochondria-associated 2 (*Aifm2*, also called *FSP1*), through the FXR or FXR-MAFG pathway^55^.

The use of doxorubicin as an antitumor agent is accompanied by cardiotoxic, hepatotoxic, and nephrotoxic side effects, and FXR has a nephroprotective effect on the doxorubicin-induced AKI model. FXR activation by dioscin, a natural saponin from various herbs, attenuates doxorubicin-induced nephrotoxicity, including oxidative stress and inflammation, by upregulating the AMP-activated protein kinase α (AMPKα) and NRF2/heme oxygenase-1 (HMOX-1) pathways^56^.

#### Role of FXR in chronic kidney diseases

Kidney fibrosis, regulated by fibroblasts and myofibroblasts, is a major event leading to progressive CKD on the way to end-stage kidney failure and a major determinant of kidney failure^57^. Inflammation and fibrosis are two CKD pathological hallmarks that involve the cytokine TGFβ^58,59^. Activated TGFβ initiates the oligomerization of the TGFβ receptor and the phosphorylation of receptor-associated Smads (R-Sma and Smads against decapentaplegics, such as Smad2 and Smad3). Then, it forms the R-Smad/common Smad (Co-Smad, Smad4) complex and translocates into the nucleus to regulate the transcription of target genes, including α-smooth muscle actin (α-SMA), collagens, and inhibitory Smad7^59^. In the mouse model of CKD via unilateral ureteral obstruction, FXR activation attenuates kidney fibrosis by suppressing SMAD3 expression but does not alter the mRNA levels of SMAD2 and SMAD4^60^. Additionally, FXR activation decreases kidney fibrosis by inhibiting Yes-associated protein 1 (YAP) signaling, which regulates fibroblast activation and extracellular matrix synthesis^61–63^. The TGFβ-induced activation of YAP accelerates kidney fibrosis by activating the expression of its target genes through YAP phosphorylation and nuclear translocation. Activation of FXR by agonists (GW4064 and WAY-362450) attenuates kidney fibrosis by inhibiting the phosphorylation of Src kinases (a family of nonreceptor tyrosine kinases) and Src-mediated activation of YAP in PT epithelial cells and interstitial fibroblast cells^62^. EDP-305, a novel FXR agonist, can also reduce kidney fibrosis by inhibiting YAP phosphorylation and translocation^63^. In the future, the antifibrotic effect of FXR in the kidneys should be studied in detail.

#### Role of FXR in diabetic nephropathy

Studies on the liver have shown that FXR activation regulates glucose metabolism through the transcriptional repression of gluconeogenesis- and glycolysis-related genes, including peroxisome proliferator-activated receptor-γ coactivator 1α (PGC-1α), phosphoenolpyruvate kinase (PEPCK), glucose-6-phosphatase (G6Pase), and fructose-1,6-bisphosphatase 1 (FBP1)^64,65^.

DN is a diabetes mellitus complication characterized by proteinuria and progressive loss of kidney function. The pathogenetic mechanism of DN is complex and driven by impaired glucose metabolism in diabetes, which triggers other metabolic defects, including the pathological activation of growth factors, the formation of high levels of advanced glycation end products, hyperglycemia, hemodynamic dysfunction, oxidative stress, proinflammatory cytokine upregulation, and the alteration of lipid metabolism^30,66^.

The importance of lipid metabolism in the pathogenesis of kidney diseases related to diabetes mellitus has been investigated. The expression levels of SREBP-1 and fatty acid synthase (FAS) increase in a rat model of streptozotocin-induced diabetes mellitus and murine cortical tubule cells treated with high glucose, resulting in increased triglyceride accumulation^30^. In Akita and OVE26 mice, which are genetic mouse models of type 1 diabetes mellitus, the expression levels of FXR, SHP, PPARα, and PPARδ decrease in the kidneys compared with those of the WT; conversely, the levels of triglyceride and cholesterol and the expression of SREBP-1c and ChREBP, which increase fatty acid synthesis, increase^29^.

In the liver, the importance of FXR as a negative regulator of SREBP-1c and ChREBP and a positive regulator of PPARα activity has been studied; FXR has also been explored for its role in reducing triglyceride and cholesterol levels^64,67–70^.

FXR agonists (GW4064 and CA) prevent the increased expression of SREBP-1 in the kidneys of high fat-diet (HFD)-induced mice, a model of diet-induced obesity and insulin resistance. In addition, the treatment of *db/db* mice with GW4064 or CA decreases the expression of SREBP-1 and profibrotic growth factors, including TGFβ, connective tissue growth factor (CTGF), and plasminogen activator inhibitor-1 (PAI-1)^32^. Visfatin (also called a pre-B-cell-enhancing factor) is an adipocytokine secreted by visceral adipocytes and is involved in the pathogenesis of DN. FXR agonist (GW4064) treatment reduces TGFβ/SMAD signaling and inflammatory responses by inhibiting increased visfatin expression in high glucose-induced human mesangial cells^71^.

To determine the pivotal role of FXR in DN, Wang XX et al. comparatively studied streptozotocin-induced nephropathy-resistant C57BL/6 FXR KO and nephropathy-susceptible DBA/2 J mice^72^. In the DN model, FXR deficiency accelerates kidney injury, and FXR agonist administration attenuates injury by decreasing proteinuria, glomerulosclerosis, and tubulointerstitial fibrosis and by regulating lipid metabolism, particularly by decreasing the expression of SREBPs^72^. In addition, the same group performed RNA sequencing in DBA/2 J mice with streptozotocin-induced hyperglycemia after these mice were treated with the FXR agonist OCA, TGR5 agonist INT-777, or dual agonist INT-767. Consequently, they found that OCA regulates the lipogenesis pathway, and INT-777 modulates mitochondrial biogenesis-related genes; INT-767 shows a combination of these effects. INT-767 prevents DN through multiple pathways, including the stimulation of AMPK-SIRT1-PGC1α-SIRT3-ERRα signaling, which regulates the expression of mitochondrial FAO-related genes and inhibits the activation of cholesterol metabolism. In addition, in a diet-induced obesity mouse model, the FXR/TGR5 dual agonist suppresses diet-induced kidney disease by changing the composition of kidney bile acids, reducing their accumulation, and decreasing triglyceride and ceramide levels in the kidney. However, this dual agonist effect cannot compensate for the absence of FXR by intact TGR5 in the absence of FXR^73^.

By using the UPLC‒MS/MS approach, a cohort study on the correlation between the composition of bile acids and diabetes risk found that unconjugated primary bile acids are associated with a reduced diabetes risk; conversely, conjugated primary bile acids are related to an increased diabetes risk^74^. Another study on the effect of the bile acid composition in DN has reported that QiDiTangShen granules, a traditional Chinese herbal medicine for the treatment of diabetic kidney disease, alter the compositions of bile acids and the gut microbiota; they also decrease urinary albumin excretion and alleviate pathological kidney injury in *db/db* mice. However, QiDiTangShen granules are not directly involved in FXR expression^75^.

A study on the importance of maladaptive ER signaling in the pathogenesis of DN has assessed the therapeutic effect of the chemical chaperone tauroursodeoxycholic acid (TUDCA) against DN. TUDCA activates FXR and induces the expression of FXR-dependent genes, including suppressor of cytokine signaling 3 (SOCS3) and dimethylarginine dimethylaminohydrolase 1 (DDAH1), thereby improving glomerular and tubulointerstitial damage in the DN mouse model. Therefore, the combined effect of TUDCA and renin-angiotensin-aldosterone system (RAAS) inhibition should be subjected to clinical studies for the treatment of patients with DN^76^. However, in this study, FXR activation by TUDCA did not show an alteration in the expression of *Shp*, which is a known FXR target gene; previous studies also revealed that TUDCA treatment does not affect *Shp* and *Bsep* expression in primary mouse hepatocytes^77,78^. According to metagenomic and metabolomic analyses of data from patients with metformin-treated type 2 diabetes, the levels of the bile acids glycoursodeoxuycholic acid (GUDCA) and TUDCA in the gut increase. In experiments involving intestine-specific FXR KO mice, GUDCA and TUDCA are endogenous antagonists of intestinal FXR^79^. Further studies should be performed on the role of TUDCA as an FXR agonist or antagonist and the correlations among the regulation of the bile acid profile, FXR activity, and renal disease.

Posttransplant diabetes mellitus is a common metabolic complication after solid organ transplantation, mainly resulting from the universal use of common immunosuppressive drugs, such as calcineurin inhibitors, especially tacrolimus (FK506). Tacrolimus-induced dysglycemia inhibits FXR expression and glucose uptake and induces gluconeogenesis; conversely, GW4064 or FXR overexpression suppresses gluconeogenesis and promotes glucose uptake by regulating the expression of PEPCK and GLUT2 in HK-2 cells and the kidneys in mice^80^. However, further studies should be conducted on the role and mechanism of FXR in the regulation of kidney glucose metabolism.

## Conclusion and future perspectives

**Fig. 1:**
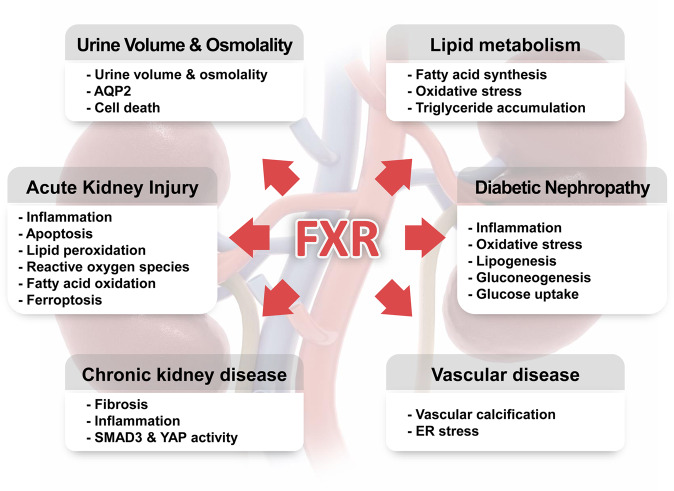
Roles of FXR in the physiology and pathophysiology of the kidney. Schematic of the roles of FXR in kidney physiology and diseases. Activated FXR in the kidney regulates urine volume, osmolality, and lipid metabolism. It has protective effects against acute kidney injury, chronic kidney disease, diabetic nephropathy, and vascular diseases through anti-inflammatory, antifibrotic, antilipogenic, and antioxidant effects. It also plays regulatory roles in glucose metabolism.

Among the global health problems, the morbidity and mortality associated with kidney diseases are increasing every year; therefore, efficient strategies should be developed to prevent and treat these diseases. Studies on the kidney-specific expression of nuclear receptors in recent decades have aided our understanding of how these factors contribute to kidney physiology and pathophysiology. In this review, one of these factors, FXR, was discussed (Fig. 1).

FXR activation by agonists has kidney-protective effects against fibrosis, inflammation, apoptosis, ferroptosis, and ROS accumulation and plays roles in lipid and glucose metabolism. However, further studies should explore the functions of kidney-specific FXR in detail. Most importantly, how FXR contributes to the regulation of kidney homeostasis in different cell types remains uncharacterized. The kidney has various cell types; although recent single-cell RNA-sequencing studies have defined the precise expression and localization of nuclear receptors, including PPARs, MR, LXRs, PXR, VDR, GR, ER, and FXR, studies investigating the functional roles of these nuclear receptors through overexpression and knockout in specific regions of the kidney are limited. Many studies have also been conducted on the physiological and pathophysiological roles of FXR PTMs, including acetylation, phosphorylation, SUMOylation, ubiquitination, glycosylation, and methylation^81^. Because PTMs often function in tissue- and context-specific manners, FXR PTMs in the kidney should be studied, although most studies have focused on FXM PTMs in the liver. In studies on FXR PTMs, the action of FXR should be targeted to a specific tissue. Since there are several cell types in the kidney, studies should also determine how to target a specific area and cell in the kidney. Therefore, related studies should focus on FXR PTMs in the kidney and the roles of each of these PTMs in various disease conditions.

Many types of therapeutic applications to modulate FXR expression and activity are being developed, and FDA-approved drugs targeting FXR for disease treatment are under clinical trials. The clinical trials and ongoing studies on FXR agonists and antagonists in different diseases are summarized in Table 2. However, FXR agonists or antagonists for the treatment of kidney diseases are currently unavailable; safe therapeutics with few side effects should be developed.

**Table 2:** Summary of the FXR modulating compounds in clinical trials.

Agonists	EC_50_/IC_50_	NCT identifiers	Clinical trial phase	Clinical indication^a^
Obeticholic acid	99 nM^82^	NCT02308111	Phase 4	PBC
		NCT0363322	Phase 4	PBC
		NCT0254835	Phase 3	NASH
		NCT03439254	Phase 3	PBC and NASH
		NCT01473524	Phase 3	PBC
		NCT05450887	Phase 3	PBC
		NCT04956328	Phase 3	PBC
EDP-305	8 nM^83^	NCT03421431	Phase 2	NASH
		NCT03394924	Phase 2	PBC
		NCT04378010	Phase 2	NASH
GW4064	65 nM^84^	n/a	n/a	n/a
Tropifexor (LJN452)	0.2 nM^85^	NCT04065841	Phase 2	NASH
		NCT02855164	Phase 2	NASH
		NCT02516605	Phase 2	PBC
		NCT03517540	Phase 2	NASH
		NCT04147195	Phase 2	NAFLD and NASH
Cilofexor (GS-9674)	43 nM^86^	NCT03890120	Phase 3	PSC
		NCT02943447	Phase 2	PBC
		NCT02781584	Phase 2	NAFLD and NASH
FXR-450 (XL335, WAY 362450)	4 nM^87^	NCT00499629	Phase 1	Safety and pharmacokinetics
Nidufexor (LMB763)	7 nM^88^	NCT03804879	Phase 2	Diabetic Nephropathy
MET409	16 nM^89^	NCT04702490	Phase 2	NASH, NAFLD and Type 2 diabetes
TERN-101 (LY2562175)	193 nM^90^	NCT04328077	Phase 2	NASH
Vonafexor (EYP001a)	37.4 nM^91^	NCT03812029	Phase 2	NASH
ASC42	n/a	NCT05190523	Phase 2	PBC
		NCT05107778	Phase 2	HBV
Antagonist				
Guggulsterone	17 μM^92^	NCT01492998	n/a	HCV

*PBC* primary biliary cholangitis, *NASH* nonalcoholic steatohepatitis, *NAFLD* nonalcoholic fatty liver disease, *PSC* primary sclerosing cholangitis, *n/a* not applicable, *HBV* hepatitis B virus, *HCV* hepatitis C virus.

^a^These studies were registered with ClinicalTrials.gov unless otherwise specified.

Genetic manipulation tools and nanomaterials for kidney-targeted drug delivery have been extensively studied in recent years and will be used to modulate the expression and activity of NRs, such as FXR, to understand the roles of these receptors and their potential as fundamental therapeutic strategies in kidney diseases.
